# Integrated Depression Care and Livelihood Interventions for Low-Income Women in Vietnam: Protocol for a Cluster Nonrandomized Controlled Trial (LIFE-DM)

**DOI:** 10.2196/80546

**Published:** 2026-06-05

**Authors:** Victoria Khanh Ngo, Thinh Toan Vu, Kunmi Sobowale, Trung Tu Lam, Tam Nguyen, Sebastian Linnemayr, Wenjing Huang

**Affiliations:** 1 RAND Corporation, Santa Monica, CA, United States; 2 Center for Innovation in Mental Health, CUNY Graduate School of Public Health and Health Policy, 55 W 125th St, New York City, NY, 10027, United States, 646-364-9600; 3 Department of Psychiatry and Biobehavioral Sciences, University of California Los Angeles, Los Angeles, CA, United States; 4 Da Nang Psychiatric Hospital, Da Nang, Vietnam; 5 Basic Needs, Hanoi, Vietnam

**Keywords:** depression, behavioral activation, problem-solving therapy, microfinance, poverty, women, Vietnam

## Abstract

**Background:**

In Vietnam, economically disadvantaged women face compounded risks due to gender inequality, financial instability, and limited access to mental health care. Community health stations (CHSs), the frontline entry point into the health system and the most accessible primary care facilities, typically lack trained mental health providers, further exacerbating an already existing treatment gap. While evidence-based treatments for depression exist, most interventions address either mental health or economic hardship separately, limiting their effectiveness in resource-constrained settings.

**Objective:**

This study aims to evaluate the effectiveness of Livelihood Integration for Effective Depression Management (LIFE-DM), an integrated intervention combining group-based psychotherapy with microfinance and livelihood support, compared with enhanced treatment as usual (E-TAU), among low-income women. We hypothesize that LIFE-DM participants will show greater program participation and improvements in mental health, psychosocial, and economic outcomes relative to E-TAU.

**Methods:**

A matched-pair cluster nonrandomized controlled trial was conducted at 4 CHSs in Da Nang, Vietnam, with 2 sites allocated to LIFE-DM and 2 to E-TAU. A total of 166 low-income women aged 18 to 55 years who screened positive for depression (9-item Patient Health Questionnaire score ≥10) were enrolled. LIFE-DM participants received group behavioral activation treatment and were offered microfinance loans, vocational training, and personal finance support. E-TAU participants were offered free antidepressant treatment and referral as usual to the Women’s Union for livelihood support. Primary outcomes are severity of depression symptoms and remission rates at 6- and 12-month follow-up. Secondary outcomes include economic variables such as income and employment status, as well as functioning, quality of life, self-efficacy, behavior activation, and program participation. The data were collected using in-person surveys, clinic logs, and program records. Analyses will follow an intention-to-treat approach using longitudinal regression models with propensity score weighting and adjustment for clustering.

**Results:**

This study was funded prior to implementation, and the data were collected from February 2014 to September 2015. Participant recruitment has been completed, with 166 women enrolled across 4 CHSs. Data analysis is in progress, and the findings are expected to be disseminated in 2027.

**Conclusions:**

This study is among the first to rigorously evaluate an integrated mental health and livelihood intervention targeting both depression and poverty among economically disadvantaged women. If effective, LIFE-DM may offer a scalable, community-based model for improving mental health and economic well-being among vulnerable women in low-resource settings.

## Introduction

Depression is the leading mental health burden globally [1], and women are particularly vulnerable, experiencing depression at rates 2 to 3 times higher than men [2]. A growing number of epidemiological studies highlight a negative spiral between poverty and mental illness [3], including depression, especially for women in low-income countries. Particularly, economic hardship increases the risk of depression through chronic stress and reduced access to resources, while depression contributes to reduced productivity, income loss, and deeper financial instability [4]. Moreover, in these settings, patriarchal cultures, restrictive gender roles, and widespread gender inequalities exacerbate the problem [5,6]. Despite the well-established link between mental health and socioeconomic factors [4], few interventions address both depression and the socioeconomic context of high-risk and economically disadvantaged communities, especially in low-resource settings.

In low- and middle-income countries (LMICs), such as Vietnam, there is a substantial treatment gap for depression, with most individuals who require care receiving little to no treatment [7-9]. LMICs bear approximately 80% of the global mental health disease burden, yet they have disproportionately limited resources to address it [10]. In Vietnam, this gap is particularly evident at the primary care level, where community health stations (CHSs), the frontline entry point into the health system, serve as the most accessible facilities but typically lack trained mental health providers and psychosocial services [11]. Effective and relatively simple treatments, such as antidepressants, and psychological interventions, such as problem-solving therapy (PST) and behavior activation (BA) for depression, exist [12,13] and have been demonstrated to be effective across various countries [14], including Vietnam [11]. However, few interventions have been scaled up in primary care settings to simultaneously and systematically address depression and the economic conditions that contribute to its persistence.

As in many other LMICs, significant barriers contribute to this treatment gap [7-9,15,16] including a shortage of mental health professionals, considerable stigma surrounding mental illness, and a lack of public understanding about depression and mental health. To increase access and availability of depression care, task-shifting (ie, training nonspecialists to deliver mental health services) and integration of mental health services into other key health sectors have been recommended [8,17]. These approaches are consistent with broader conceptual frameworks, such as the social determinants of health and collaborative care models [18], which emphasize addressing both clinical symptoms and underlying economic drivers of poor mental health outcomes.

The World Health Organization recognizes mental health as a development issue in LMICs [19], but no interventions have specifically integrated depression care and microfinance services. Microfinance, financial services, and products for low-income individuals have been heralded as a promising poverty alleviation strategy and gained widespread community adoption in LMICs, particularly for women [20-22]. Microfinance interventions typically include small loans, savings programs, microgrants, financial literacy, or livelihood training aimed at increasing income-generating activities and financial resilience [23,24]. However, they may also introduce pressures related to profit generation and loan repayment [25]. A few studies have examined whether microfinance interventions affect depression, yielding mixed findings. Studies in Uganda [26] and the Democratic Republic of Congo found no effect on depressive symptoms [27], while a study in South Africa revealed some reduction in symptoms in men but not in women [28]. These mixed results suggest that microfinance interventions alone may be insufficient to address the psychological and behavioral mechanisms underlying depression.

From a conceptual perspective, integrating microfinance services into depression care may interrupt the poverty-depression cycle through multiple pathways. Psychotherapy, such as BA or PST, could increase engagement with adaptive, rewarding activities [14] and strengthen problem-solving skills to handle life problems and future challenges [29]. Additionally, livelihood support reduces financial stress and enhances economic stability [24]. Together, these components may increase the relevance and uptake of depression care in socioeconomically constrained settings. In addition, skills learned in these approaches can be directly applied to managing financial and livelihood challenges. Despite these potential synergies, little is known about how to best integrate these services and the evidence for integrating depression care with microfinance services is largely unknown [30].

To fill this gap, we developed an integrated model of care, called Livelihood Integration for Effective Depression Management (LIFE-DM), which addresses both depression and livelihood concerns to increase treatment engagement and depression treatment effectiveness for low-income women with depression in Vietnam. This study aims to evaluate the effectiveness of LIFE-DM compared with enhanced treatment as usual (E-TAU) in improving participants’ mental and psychosocial outcomes as well as economic aspects among economically disadvantaged women with depression in Vietnam. Specifically, we hypothesized that the LIFE-DM program will (1) enhance treatment engagement; (2) improve mental health outcomes; (3) increase economic well-being (eg, family income, savings, employment); (4) improve functioning and quality of life; (5) increase self-efficacy, social connections, and behavioral skills; and (6) reduce stigma in the LIFE-DM intervention group compared to the E-TAU group at both 6- and 12-month follow-ups.

## Methods

### Intervention Development

The LIFE-DM intervention was developed through a process of adaptation, stakeholder engagement, and pilot testing. The psychotherapy component was adapted from the Community Partners in Care project [31], which includes modules on cognitions, behaviors, and social skills. Based on our formative work through the multicomponent collaborative care model for depression and stakeholder input regarding feasible approaches for task-sharing in Vietnam [32], the team selected only BA and social skills or support modules, prioritizing pragmatic, concrete skills suitable for delivery by nonspecialist providers.

A review of microfinance interventions identified five components for integration with depression care: (1) livelihood assessment, (2) livelihood planning, (3) livelihood goal setting, (4) incorporation of financial stressors into psychoeducation, and (5) integration of livelihood activities into BA exercises. The adapted curriculum was reviewed with Da Nang Psychiatric Hospital staff and CHS workers for content relevance, cultural appropriateness, and delivery feasibility.

Two pilot groups were conducted over 4 months at 2 CHSs under psychiatrist supervision to refine content and delivery. Each group completed 12 sessions, with group sizes ranging from 6 to 10 and 4 to 9 participants, respectively (21 participants total across both groups). The microfinance loan component was not included during piloting. Attendance and 9-item Patient Health Questionnaire (PHQ-9) scores were collected to assess symptom change. The pilot groups also served as the primary training vehicle for local clinical supervisors.

Participant and supervisor ratings were collected throughout the pilot phase to evaluate intervention quality. At every session, participants rated the intervention on a 3-point Likert scale across domains of understanding (eg, “the session was easy to understand”), relevance (eg, “the lessons in this session are relevant to me”), helpfulness (eg, “I found this session useful”), feeling heard and engaged by facilitators (eg, “the facilitator understood my problems”), and skill development (eg, “this session taught me how to take small steps toward my livelihood goals”). Supervisors rated sessions on adherence across multiple domains, including group facilitation (eg, set the agenda), tracking (eg, checked homework), and session-specific content (eg, reviewed lessons from previous sessions), as well as overall quality (eg, the session was structured).

Participant ratings indicated high perceived relevance and understanding, and PHQ-9 scores showed preliminary symptom improvement. Based on these pilot findings, the intervention protocol was finalized for the main trial without major modifications. Pilot participants were not included in the main trial sample.

### Description of Intervention Conditions

#### LIFE-DM Intervention

LIFE-DM is a 12-session group therapy. BA and PST focused on mood management and livelihood problems. The first 8 sessions are delivered once a week, and the remaining 4 sessions are delivered every other week, with the program usually lasting 4 to 5 months. BA is an evidence-based treatment that focuses on increasing pleasurable and meaningful behavior in order to improve mood. The theory underlying BA is that depression is maintained by environmental and behavioral factors (eg, a lack of appropriate social skills) that decrease pleasant or rewarding activities. Therefore, BA aims to improve mood by increasing engagement in positive activities and reducing activities that sustain depressed mood or hinder adaptive activities. This is achieved through techniques, such as mood monitoring, activity scheduling, and engagement in pleasurable and healthy activities [33]. PST, a cognitive-behavioral approach, provides a structured and positive method for tackling real-life challenges [34]. PST empowers individuals to better understand and manage their problems by teaching them how to accurately define and prioritize problems, brainstorm effective solutions, and take actionable steps to solve them.

In LIFE-DM, BA and PST are integrated with livelihood training. All microfinance services were provided by the Vietnamese Women’s Union (WU), which has been the primary distributor of microfinance initiatives in Vietnam since 1987, serving as the interface between banks and impoverished women. The WU has significant expertise in implementing microfinance programs and has been supported internationally by the World Bank, AusAID, the International Women’s Development Agency, and the Vietnamese government. In Da Nang, WU has operated a microfinance program since 1987 with over 60 trained staff.

The main aims of livelihood training in the LIFE-DM program are to develop participants’ vocational and business management skills, and to build savings. Women can also receive microcredit loans to start or improve their small businesses, such as selling sugarcane drinks, setting up noodle stalls, and so forth. The microcredit program uses a group-delivered format but does not require lending group members to collectively coguarantee loan repayment, which is often a requirement in other group-based microlending programs [35]. After learning about the loan program, members can apply for a loan and complete a livelihood plan, where they indicate how they will use the money. The WU assesses the livelihood plan’s viability and determines loan eligibility. A spouse or family members can serve as a loan guarantor. Loans in the LIFE-DM program are for 3,000,000 VNĐ (US $136) with an interest rate of 0.65% per month (the interest rate used by the Bank of Social Policy for the poor) and a monthly repayment schedule of 20 months. Loan recipients are also required to contribute 15,000 VNĐ (US $0.68) each month to their savings program. At the end of the 20 months, they will receive US $15 in savings. Members attend LIFE-DM group meetings, where they learn skills to manage their stress and discuss issues related to family, social, and livelihood stressors, including difficulties with their finances, business, and loan repayment. Groups may elect to start a group rotating credit, where they can each contribute a specified amount to create a credit fund that can be rotated for each member. After the end of the 12-session LIFE-DM program, women are encouraged to continue to meet monthly to support each other in their livelihood plan and collect the loan repayment.

#### Training and Supervision

LIFE-DM was jointly provided by nurses from the CHS and community lay workers employed by the WU, with CHS nurses leading the depression content and WU lay facilitators leading the livelihood portion of the sessions. All providers’ clinical competency for the project was developed and maintained through (1) initial trainings and booster workshops, (2) direct supervision from hospital psychiatrists and psychologist supervisors, (3) guided practice group facilitation, and (4) a tiered supervision model. Training and workshops covered a range of topics, including the collaborative care model, depression, screening and assessment, stress management, BA, basic counseling, and patient engagement skills. The training included a combination of didactic presentations, role-plays, demonstrations, case consultations, and conceptualization. Training was implemented at two levels: (1) training of supervising psychiatrists and (2) training of CHS and WU staff who served as the LIFE-DM facilitators.

At the start of the study, the CHS and WU providers for the LIFE-DM program (8 facilitators: 4 CHS nurses and 4 WU community workers) participated in an initial 3-day training on collaborative care, depression care, microfinance, and the LIFE-DM protocol, delivered by the principal investigator (PI) and supported by a Da Nang psychiatrist and WU supervisors. Following the initial workshop, the LIFE-DM providers conducted a practice pilot group (from development or adaptation phase), where supervising psychiatrists provided weekly on-site supervision at CHS sites and directly supported facilitation (sometimes cofacilitating challenging sessions) and provided direct feedback on adherence and patient engagement after each session. LIFE-DM providers also received feedback from participants and reviewed feedback with supervisors at the end of each session during the pilot training phase.

Once the training group was completed, LIFE-DM providers continued to receive supervision support from Da Nang psychiatrist supervisors (who were later replaced with newly trained bachelor-level psychologist supervisors), who also attended LIFE-DM sessions, rated all sessions on adherence and quality, and provided feedback based on these ratings. Group supervision with Da Nang Psychiatric Hospital supervisors occurred weekly initially and reduced to biweekly once competence was achieved. Sessions were videotaped for supervision and were reviewed by supervisors when they did not attend sessions. The study PI also reviewed these recordings intermittently to conduct independent adherence and quality checks and support supervision of study supervisors (2 psychiatrists and 2 psychologists from Da Nang Psychiatric Hospital), which occurred weekly with the study PI during the training period and approximately twice a month for the first 2 groups of the intervention evaluation, and later once a month or on an as-needed basis.

#### Adherence and Quality Assessment

All LIFE-DM group sessions were videotaped for adherence and quality ratings by supervisors, who either rated live sessions or reviewed videotape recordings of the sessions. Adherence ratings involved an evaluation of session components, such as check-ins, agenda setting, homework review, teaching of specific skills such as mood tracking, homework assignments, and feedback, on a 4-point scale (did not deliver or discuss, poor, average, and excellent) [11,36,37]. The quality scale assesses the overall quality of the session’s structure, clarity, group engagement, and focus on skill-building. This was rated on a 3-point scale ranging from poor (1) to excellent (3).

#### Enhanced Treatment as Usual

The Vietnam National Mental Health Initiative has implemented the national depression program, where free guideline antidepressants are offered at the commune level through selected CHSs across the country. Usual care at CHSs in Vietnam typically does not include depression care; therefore, CHS sites that received training through the study and provided antidepressant treatment are considered enhanced usual care. Participants in the E-TAU CHSs are offered medication treatment, which includes fluoxetine and amitriptyline, based on a medication algorithm outlined by the Vietnam National Mental Health Program, the highest standard of public sector community mental health available nationwide. CHSs may also refer patients with social needs to the local WU or other community-based organizations, such as the Farmer’s Union, where microfinance loans and livelihood support are also available. Medication assessment and management in both LIFE-DM and E-TAU CHSs were provided by physician assistants or nurses, supported by the Da Nang Psychiatric Hospital as recommended in the national program.

### Study Site Characteristics and Recruitment

The study was conducted at CHSs, which are the most accessible and basic health care setting in Vietnam. They are the lowest administrative level in the health care system, serving a catchment area of approximately 10,000 people each. CHSs provide nonspecialized health care and refer to provincial hospitals for more complex medical care. They are generally managed by general practitioners and staffed by nurses, physician assistants, and midwives. CHS personnel typically have no training in mental health services. For the LIFE-DM program, the CHS collaborated with the Vietnamese WU, which is a well-established quasi-governmental organization focused on promoting the well-being and livelihood of Vietnamese women throughout the country. Four CHSs previously involved in the national mental health program, which offered free antidepressant medications in line with guidelines, were recruited for the study. These 4 CHSs were also similar in a number of key characteristics, including commune-level socioeconomic status (serving a high percentage of individuals considered poor to near poor according to government assignment), number of providers (4‐6 staff), composition of staff (usually run by a physician or physician assistant and supported by 3‐5 nurses), and clinic size (ie, approximately 500‐1000 patient load). Two were allocated to LIFE-DM intervention, and 2 were allocated to E-TAU.

### Participant Recruitment

Women residing in Da Nang, Vietnam, were recruited using multiple methods. Women approaching the 4 participating CHSs for health concerns were assessed for program eligibility during enrollment periods of 1 week at the start of each group. In addition, women seeking support for life stressors or exhibiting emotional distress were also referred by CHS or WU staff to be screened for the study during enrollment periods. Study inclusion criteria included (1) female gender, (2) aged 18 to 55 years, (3) monthly family income of less than 2,000,000 VNĐ (roughly US $91) per family member, (4) positive screen for depression using the PHQ-9 [38] (score of 10 or higher), and (5) no significant physical health problems or disabilities (eg, heart disease, cancer, chronic pain, blindness). Exclusion criteria include (1) positive screen on alcohol use (measured by the Alcohol Use Disorders Identification Test) [39], (2) elevated suicide risk, and (3) active psychotic and manic symptoms. Such patients were referred to the Da Nang Psychiatric Hospital for services.

### Study Design

A matched-pair cluster nonrandomized controlled trial was conducted, comparing the LIFE-DM intervention with E-TAU. Two CHSs were allocated to the LIFE-DM group intervention and 2 to the E-TAU arm, which offered guideline-based antidepressant medications. Cluster-level randomization was not feasible for two reasons: (1) the small number of available sites limited the use of randomization for achieving balance and (2) the WU, whose infrastructure was essential for delivering the microfinance component, imposed operational constraints on random assignment of microfinance services across sites. To strengthen comparability in the absence of randomization, CHSs were matched on key characteristics prior to allocation (see the *Study Site Characteristics and Recruitment* section), and within each matched pair, 1 site was assigned to LIFE-DM and the other to E-TAU.

The trial was designed to approximate real-world implementation conditions under which the program would be disseminated, in order to assess the feasibility and effectiveness in routine care settings. Although propensity score matching will be used to reduce baseline imbalances, we acknowledge that selection bias may still be present due to the nonrandomized cluster allocation and potential unmeasured confounding at both individual and cluster levels.

### Power Analysis

The primary outcomes are treatment engagement, depression symptom change in PHQ-9 score, and depression remission on the Mini International Neuropsychiatric Interview (MINI). We compare the means in a regression framework, after taking into account the design effect induced by clustering, to determine the power when comparing the intervention group to the control group. We note that the end-status analysis assumed for the power calculations can be conservative because it does not use all 3 waves of data and uses only the projected retained sample at month 12. For the power analyses, we used the conservative estimate of the projected retention rate of 79% at the 12-month follow-up. This translates to approximately 64 patients per condition at the 12-month follow-up. A recent meta-analysis found an effect size of 0.78 favoring therapies using BA over control conditions using professionals [40]. However, given the use of paraprofessionals with limited mental health training, we expect smaller effect sizes. For these power analyses, we used conservative effect sizes of 0.3 and 0.4. Since patients are clustered within intervention groups, patients’ outcomes may be correlated, even though other randomized mental health studies have found intraclass correlation coefficients that are essentially zero [41]. Using this effect size, a power of 0.85, a conservative intracluster correlation of 0.1, a cluster size of 10, and a design effect of at least 2, we determined that 142 participants were needed for the study. Calculations were performed using sampsi and sampclus commands on STATA [42]. To account for loss to follow-up, we planned to enroll 160 and ended up with a final sample size of 166 participants.

### Outcome Measures

All study measures (Table 1) were adapted using a consensus approach to ensure linguistic and cultural appropriateness for use in Vietnam. Primary outcomes focused on depression severity and diagnosis, measured by the PHQ-9 [38], which assesses depressive symptom severity, and severe mental illness screener, which was based on items from the MINI [43]. Secondary outcomes included program participation indicators, such as group attendance, engagement in livelihood activities, and acceptance of depression care. The data were gathered through face-to-face surveys, clinic logs, and program records.

**Table 1.** Main outcome measures.

Indicator	Scale or measure	Data source
Depression severity	Patient Health Questionnaire	Surveys at baseline, 6 months, and 12 months
Anxiety severity	Generalized Anxiety Disorder	Surveys at baseline, 6 months, and 12 months
Health functioning	Short-form Health Survey	Surveys at baseline, 6 months, and 12 months
Quality of life	Quality of Life Enjoyment and Satisfaction Questionnaire	Surveys at baseline, 6 months, and 12 months
Behavioral activation	Brief Activation for Depression Scale–Short Form	Surveys at baseline, 6 months, and 12 months
Social capital	Short Social Capital Assessment Tool	Surveys at baseline, 6 months, and 12 months
Program participation	Group attendance, engagement in livelihood activities, and acceptance of depression care	Clinic logs or program records

Anxiety symptoms were assessed using the 7-item Generalized Anxiety Disorder 7 scale (GAD-7) [44]. Health functioning was measured by the Short Form-12 Health Survey [45], capturing both physical and mental health status. Quality of life was assessed with the Quality of Life Enjoyment and Satisfaction Questionnaire [46], while BA was evaluated using the Brief Activation for Depression Scale–Short Form [47], which captures levels of engagement in goal-directed activities. Social capital was measured using an adapted version of the Short Social Capital Assessment Tool [48], which includes indicators of community participation and support. Economic and employment outcomes included measures of work status, income, household assets, loan and savings activities, and perceived performance of small businesses. Additional sociodemographic covariates such as age, marital status, education, mental health literacy, household size, and number of dependents were also collected to adjust for potential confounders.

### Data Analysis

The data will be analyzed with an intention-to-treat approach for all outcomes. We will conduct a longitudinal regression analysis [49] to compare the two interventions on (1) primary outcomes, including depression scores based on the PHQ-9 and remission from depression (based on MINI diagnoses), and (2) secondary outcomes, including BA, functioning, quality of life, treatment acceptance, and economic outcomes. To address internal validity concerns of a nonrandomized design, we will use propensity score-based weighting, which is a recommended statistical technique to equate groups on a large number of baseline covariates to adjust for potential baseline differences and strengthen the inferential pathway toward causation in nonrandomized design [50-52]. Propensity score weights will be calculated using baseline demographic and clinical characteristics such as the PHQ-9, the GAD-7, physical health functioning, mental health functioning, BA, social support, self-efficacy, mental health literacy, age, education, economic status, work status, marital status, and loan exposure.

A regression analysis [49] with propensity weights will be conducted to estimate treatment effect with repeated measures of our outcomes at 3 time points accounting for demographic covariates, such as age, marital status, baseline income variables, work status, baseline mental health, functioning, and social predictors. Effects may vary over 6 and 12 months; therefore, we will also test for linear time trends in the regression models. Outcomes for patients enrolled from the same intervention group, cohort, and site may be correlated. Ignoring this clustering effect would lead to the underestimation of the variance of treatment effects. Thus, we will adjust for group clustering to account for the correlation among the measures of patients enrolled in the same intervention group, cohort, and site [53].

We will examine variable distributions from all time points to assess missing data patterns within each study wave and replace them with information gathered from clinic logs and program records. Based on previous similar studies in Vietnam in general and Da Nang in particular, we expect response rates of approximately 80% at 6-month and 12-month follow-ups, resulting in about 128 patients at the 2 follow-ups. All planned analyses will use likelihood methods, which are effective under missing at random assumptions [54]. This approach helps account for participants dropping out of the study while making the most efficient use of all the data collected. Because time will be a random effect within the mixed model analyses, participants without complete data will be included fully in the analyses. For all outcomes, Cohen *d* will be calculated to estimate the effect size using pooled SD of baseline scores. Two-sided *P*<.05 will be considered statistically significant.

### Ethical Considerations

This study was approved by the Da Nang Psychiatry Hospital Institutional Review Board (01/17/HDDD) and the RAND Corporation (2012‐0031 RAND IRB). The study was conducted in accordance with the Declaration of Helsinki. All participants provided written informed consent before participating in the study. Participants received 50,000 VNĐ (approximately US $2.30) for completing each survey as compensation for their time. All participant data will be deidentified and assigned unique study identification numbers prior to analysis.

## Results

### Participant Flow

**Figure 1.**
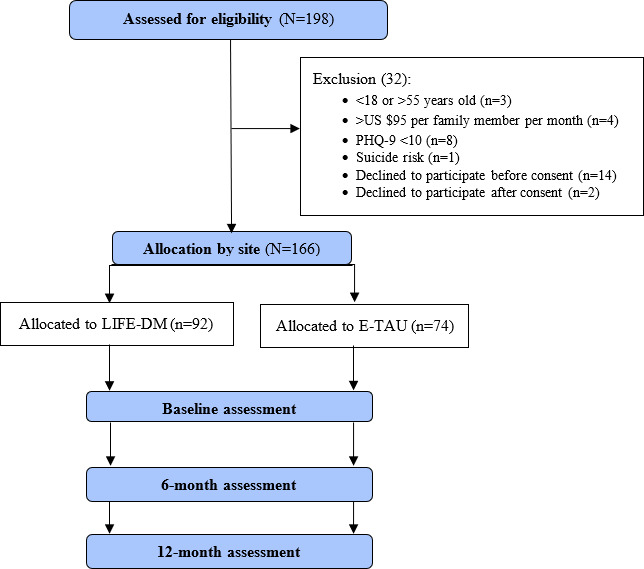
LIFE-DM trial flow chart. E-TAU: enhanced treatment as usual; LIFE-DM: Livelihood Integration for Effective Depression Management; PHQ-9: 9-item Patient Health Questionnaire.

This study was funded prior to implementation, and the data were collected from February 2014 to September 2015. A total of 198 women were screened for eligibility, of whom 166 met the inclusion criteria and were enrolled. Thirty-two women were excluded for the following reasons: outside the eligible age range (n=3), income above the threshold (n=4), PHQ-9 score below 10 (n=8), elevated suicide risk (n=1), declined participation prior to consent (n=14), or withdrew after consenting (n=2; Figure 1). Baseline characteristics of participants are presented in Table 2. Data analysis is in progress, and the findings are expected to be disseminated in 2027.

**Table 2.** Participants’ baseline characteristics and group differences^a^.

Indicators	Overall (N=166)	E-TAU^b^ (n=74)	LIFE-DM^c^ (n=92)	*P* value
Demographics				
Age, mean (SD)	42.27 (8.37)	42.72 (8.59)	41.91 (8.23)	.54
Marital status (currently married), n (%)	119 (71.69)	53 (71.62)	66 (71.74)	.99
Size of household, mean (SD)	4.43 (1.53)	4.43 (1.46)	4.42 (1.59)	.97
Number of children, mean (SD)	2.29 (1.04)	2.24 (1.06)	2.33 (1.04)	.61
Number of children ages 5‐18 (y), mean (SD)	1.14 (0.91)	1.18 (0.87)	1.11 (0.94)	.64
Reading ability (can read easily), n (%)	135 (81.33)	56 (75.68)	79 (85.87)	.094
Writing ability (can write easily), n (%)	131 (78.92)	53 (71.62)	78 (84.78)	.04
Education level (completed primary school), n (%)	122 (73.49)	47 (63.51)	75 (81.52)	.009
Economic factors				
Home ownership, n (%)				.46
Does not own home	60 (36.14)	29 (39.19)	31 (33.70)	
Owns home	106 (63.86)	45 (60.81)	61 (66.30)	
Monthly income (US $), mean (SD)				
Family income	169.94 (82.10)	166.36 (87.74)	172.82 (77.65)	.62
Individual income	82.35 (53.83)	71.1 (54.40)	91.83 (51.78)	.02
Household per capita income	40.52 (18.93)	39.42 (20.02)	41.41 (18.07)	.50
Poverty (World Bank threshold), n (%)	49 (29.52)	27 (36.49)	22 (23.91)	.08
Employed, n (%)	120 (81.63)	39 (68.42)	81 (90.00)	<.001
Owned business in past, n (%)	68 (40.96)	18 (24.32)	50 (54.35)	<.001
Currently owns business, n (%)	59 (35.54)	16 (21.62)	43 (46.74)	<.001
Currently has loans, n (%)	79 (47.59)	37 (50.00)	42 (45.65)	.58
Current loan amount (US $), mean (SD)	700.12 (1083.26)	482.95 (693.33)	890.80 (1315.01)	.10
Currently saves money, n (%)	31 (18.67)	12 (16.22)	19 (20.65)	.47
Current amount saved (US $), mean (SD)	189.85 (410.87)	337.88 (628.18)	91.16 (82.86)	.11
Mental health				
PHQ-9^d^ score (0‐27), mean (SD)	14.43 (3.46)	14.28 (2.95)	14.54 (3.83)	.63
Currently have depression (MINI^e^), n (%)	141 (85.45)	57 (78.08)	84 (91.30)	.02
Had depression in the past (MINI), n (%)	110 (66.67)	45 (61.64)	65 (70.65)	.22
GAD-7^f^ (0‐21), mean (SD)	9.58 (3.52)	9.03 (2.79)	10.01 (3.96)	.08
Psychosocial				
BADS-SF^g^ (0‐54), mean (SD)	21.02 (3.99)	20.81 (4.08)	21.20 (3.92)	.54
Experienced domestic violence, n (%)	44 (26.51)	21 (28.38)	23 (25.00)	.62
Q-LES-Q^h^ (0‐100), mean (SD)	28.83 (9.18)	27.99 (8.69)	29.50 (9.55)	.30
MOS-SF-12^i^ physical component, mean (SD)	36.77 (5.28)	36.94 (4.87)	36.63 (5.61)	.71
MOS-SF-12 mental component, mean (SD)	27.33 (7.05)	27.42 (6.10)	27.25 (7.77)	.88
MOS-SF-12 total (0‐100), mean (SD)	35.06 (22.10)	32.6 (23.95)	37.05 (20.40)	.20
Structural social capital support (including both active group and social support), n (%)				.04
None	54 (33.96)	32 (44.44)	22 (25.29)	
One	36 (22.64)	15 (20.83)	21 (24.14)	
Two or more	69 (43.40)	25 (34.72)	44 (50.57)	
Structural social capital (active group membership only), n (%)				.06
None	92 (55.42)	48 (64.86)	44 (47.83)	
One	61 (36.75)	23 (31.08)	38 (41.30)	
Two or more	13 (7.83)	3 (4.05)	10 (10.87)	
Business self-efficacy (0‐10), mean (SD)	6.26 (1.51)	5.89 (1.45)	6.56 (1.50)	.005
Goal self-efficacy (4-20), mean (SD)	9.74 (2.28)	9.23 (2.07)	10.15 (2.38)	.01
Structural barriers to care, mean (SD)	4.27 (2.19)	4.78 (1.88)	3.87 (2.35)	.008
Mental health literacy, n (%)	72 (43.37)	28 (37.84)	44 (47.83)	.20
Depression stigma, mean (SD)	26.17 (3.00)	25.81 (3.60)	26.47 (2.39)	.17
Patient satisfaction, n (%)				
With CHS^j^	17.98 (1.13)	17.98 (0.97)	17.98 (1.25)	.99
With Women’s Union	18.13 (1.33)	17.69 (0.48)	18.29 (1.51)	.17

a The data in this table are not adjusted for survey design. Numbers do not all sum to 166 because of item nonresponse or not applicable. The percentages are not all 100% due to rounding.

b E-TAU: enhanced treatment as usual.

c LIFE-DM: Livelihood Integration for Effective Depression Management.

d PHQ-9: 9-item Patient Health Questionnaire.

e MINI: Mini International Neuropsychiatric Interview.

f GAD-7: 7-item Generalized Anxiety Disorder.

g BADS-SF: Behavior Activation for Depression Scale Short Form.

h Q-LES-Q: Quality of Life Enjoyment and Satisfaction Questionnaire.

i MOS-SF-12: 12-item Medical Outcomes Study Short Form.

j CHS: community health station.

### Baseline Characteristics

Among the 166 participants enrolled in the study (Table 2), the average age was 42 (SD 8) years. Most women were married (n=119, 71.69%). The majority of participants were able to easily read (n=135, 81.33%) and write (n=131, 78.92%), and 73.49% (n=122) completed primary school. Most women were employed (n=120, 81.63%). On average, the per capita family income was US $40.52 (SD 18.93) per month, and individual income was US $82.35 (SD 53.83) per month. In addition, 29.52% (n=49) lived below the poverty level according to World Bank 2010 criteria for Vietnam (653,000 VNĐ per person per month, or US $1 per person per day). Approximately 47.59% (n=79) reported having existing loans.

The mean score on the PHQ-9 scale for depression was 14.43 (SD 3.46), and 9.58 (SD 3.52) on the GAD-7 scale for anxiety. Most participants had a current diagnosis of depression (n=141, 85.45%). In terms of social capital support, 55.42% (n=92) of the participants had no active memberships in any social groups in the community, and 33.96% (n=54) of the participants reported having no source of social support (including both organizations and individuals). Participants reported an average of 4.27 (SD 2.19) structural barriers to care, such as transportation, money, and child-care responsibilities. In addition, participants reported a mean depression stigma score of 26.17 (SD 3; possible range is 0-40), where higher scores indicate higher perceptions of stigma.

In terms of economic characteristics, the groups differed with regard to business ownership and employment status, with women in the intervention group more likely to have prior experience owning a business (n=50, 54.35% vs n=18, 24.32%; *P*<.001), currently owning a business (n=43, 46.74% vs n=16, 21.62%; *P*<.001), and currently working (n=81, 90% vs n=39, 68.42%; *P*<.001). Those in the intervention group had higher individual income (US $91 per month vs US $71 in the control group; *P*=.02), but there were no significant differences between the groups by family income.

In terms of mental health characteristics, the intervention group was found to have a higher percentage who met the criteria for depression (n=84, 91.30% vs n=57, 78.08%; *P*=.02) according to the MINI Diagnostic Interview. However, severity scores on the PHQ-9 did not differ between the 2 groups. Participants in the intervention group also reported a lower mean number of structural barriers to care (3.87 vs 4.78; *P*=.008) and higher goal self-efficacy (10.15 vs 9.23; *P*=.01), as well as business self-efficacy (6.66 vs 5.74; *P*=.005).

## Discussion

### Study Rationale and Implications

The LIFE-DM trial aims to evaluate whether an integrated depression care and livelihood intervention can increase treatment engagement and is more effective than E-TAU in a collaborative care approach. The study addresses a critical gap in global mental health: how to effectively deliver evidence-based depression care to populations burdened by both mental illness and poverty. Despite the strong relationship between depression and poverty [3], this combined approach to care has not been previously evaluated in a controlled study. Many individuals in resource-poor settings face barriers to mental health care [15,16], including lack of awareness and knowledge about depression and depression care, stigma about mental health treatments, cost, economic stressors, and lack of value placed on mental health treatment. The LIFE-DM model is designed to integrate psychosocial support with financial empowerment, through microfinance activities, vocational training, and savings programs, to increase the acceptability and perceived value of depression treatment. This integrated approach is hypothesized to improve mental health, functional status, and economic well-being. If effective, this model may offer a scalable approach for similar low-resource settings.

This model is informed by principles of “stepped care” and “community-partnered process,” which emphasize accessible, acceptable, and contextually grounded interventions [55]. By incorporating income-generating activities, vocational support, and microfinance opportunities, the intervention may help reframe depression care as a means of improving broader life circumstances, thus reducing stigma and enhancing uptake [22]. It is anticipated that this integrated approach will increase the perceived relevance and acceptability of mental health interventions, especially among populations that view mental illness through social and economic lenses rather than clinical ones.

Furthermore, our findings are expected to contribute to the growing field of task-shifting in global mental health [17]. By engaging paraprofessional providers, WU, and CHS staff, the intervention explores the potential for extending the collaborative care models to settings with limited specialist resources. This approach aligns with broader efforts to strengthen health systems through decentralization, community-based services, and the use of nonspecialist providers. The findings from this study may help inform the development of scalable models for delivering evidence-based depression care in resource-limited contexts.

### Limitations

This study has several limitations. The lack of randomization was the primary limitation of the study due to the pragmatic design of the trial. As the baseline data show, there were a number of baseline mental health and economic differences between the LIFE-DM group and the E-TAU group. Although both groups were low-income and had elevated levels of depressive symptoms, the LIFE-DM intervention group reported a higher rate of business experience and ownership as well as higher individual income. They also tended to have higher levels of depressive symptoms but no differences in the rate of depression than the E-TAU group. This suggests that there may be some level of selection bias particularly as referrals for LIFE-DM appear to be motivated by interest in the microfinance program. As these are common sample bias issues in pragmatic trials, we will statistically equate groups using propensity weights, which is a recommended solution for pragmatic or real-world trials where randomization may not be possible. However, residual confounding may remain due to unmeasured differences between the groups. We therefore interpret the findings as estimates of effectiveness under real-world conditions rather than causal effects equivalent to those derived from randomized controlled trials. A further limitation is that the study was conducted in only 4 urban community health stations in Da Nang, which may limit generalizability to rural or other low-resource settings. Finally, reliance on self-reported measures may introduce bias, particularly for sensitive outcomes such as income and mental health symptoms.

### Conclusions

The LIFE-DM trial is designed to examine an integrated approach to addressing the dual burden of depression and poverty in low-resource settings. By integrating mental health care with economic empowerment and leveraging community-based human resources, the intervention has the potential to offer a scalable, contextually relevant model that may be adaptable across diverse settings. As global health systems seek sustainable, multifaceted solutions to the dual burdens of mental illness and poverty, LIFE-DM may help inform future policy, programming, and research efforts, including potential integration into Vietnam’s National Health Insurance scheme through reimbursement mechanisms for community-based psychosocial care delivered in primary care settings. Ongoing analyses are expected to provide further insight into its effectiveness and feasibility for broader implementation.

## Abbreviations

BA: behavioral activation
CHS: community health station
E-TAU: enhanced treatment as usual
GAD-7: 7-item Generalized Anxiety Disorder
LIFE-DM: Livelihood Integration for Effective Depression Management
LMIC: low- and middle-income country
MINI: Mini International Neuropsychiatric Interview
PHQ-9: 9-item Patient Health Questionnaire
PI: principal investigator
PST: problem-solving therapy
WU: Women’s Union
